# How to Perform a Completely Fluoroless Ablation

**DOI:** 10.19102/icrm.2023.14055

**Published:** 2023-05-15

**Authors:** Hussam Abuissa, Attila Roka

**Affiliations:** ^1^Department of Clinical Cardiac Electrophysiology, Creighton University School of Medicine, Omaha, NE, USA

**Keywords:** Atrial fibrillation, cryoablation, fluoroless ablation, supraventricular tachycardia, ventricular tachycardia

## Abstract

Radiation exposure related to electrophysiology catheter ablation procedures carries small but non-negligible stochastic and deterministic effects on health. Lead aprons can also place considerable pressure on the spinal column, resulting in potentially detrimental consequences. Fortunately, however, advancements in tools used for arrhythmia mapping and ablation have made it feasible to reduce or essentially eliminate the need for fluoroscopy, with no impact on the efficacy or safety of such procedures, as demonstrated by several long-term outcome studies. In this review, we describe our stepwise approach to safely and efficiently perform a completely fluoroless ablation.

## Introduction

Fluoroscopy has traditionally been the cornerstone of ablation procedures for cardiac arrhythmias. However, several studies have demonstrated the adverse effects of radiation, including skin injury, cataract, decreased fertility, and malignancies.^1–3^ Additionally, there is a significant burden of orthopedic injuries resulting from the regular use of lead protective apparel.^4,5^ Such issues have piqued the interest of many electrophysiologists to reduce or even eliminate radiation exposure, especially as the number of ablation procedures has been on the rise. Over the last several years, technological advancements, especially in electroanatomic mapping and intracardiac echocardiography (ICE), have made it feasible to achieve such a goal^6–8^ without compromising safety, efficacy, or procedural duration.^9–11^

This review describes our stepwise approach to safely and efficiently perform a completely fluoroless ablation, which should be the new standard of care.

## Line placement

Placing lines is one of the most critical steps in the fluoroless ablation procedure. If all lines are placed in the right groin, short sheaths are typically used; however, if both sides of the groin are used for access, then using long sheaths, especially on the left side, is a safer approach so that catheters can be advanced into the inferior vena cava (IVC) and subsequently into the heart. A micropuncture needle set, in conjunction with bedside ultrasound if needed, is used for access. The micropuncture wire is then exchanged for a J-tip wire, which needs to be advanced beyond the anticipated depth of the sheath. Should any difficulty be encountered, a Wholey guidewire (Covidien, Dublin, Ireland) should be used. Continuous electrocardiographic monitoring is recommended, as a mechanically induced arrhythmia may be evident if the wire is advanced too far into the right atrial appendage or right ventricle.

## Catheter placement and electroanatomic mapping

We start by placing a multipolar catheter capable of electroanatomic mapping and matrix buildup (PentaRay NAV ECO high-density or DECANAV catheter; Biosense Webster, Diamond Bar, CA, USA) or an ablation catheter, depending on the specific ablation case. The catheter is typically advanced via one of the right groin sheaths until it can be visualized by the mapping system. Anterior catheter direction may lead from the groin area to the heart with no intermediate stops, as the anterior aspect of the IVC has no collateral vessels. Should any difficulty be encountered in the groin area, switching to a long sheath would typically resolve the issue. Advancement is continued until an electrogram is detected, which indicates that the right atrium has been reached. We keep advancing cranially while maintaining septal positioning into the superior vena cava (SVC), which is demarcated by the loss of electrograms. The mapping catheter is then withdrawn back into the right atrium and deflected to be advanced anteriorly with a clockwise rotation into the right ventricle as guided by the electroanatomic map. The His-bundle location is marked, and the mapping catheter is then withdrawn posteriorly and inferiorly while maintaining septal positioning until it falls into the coronary sinus as confirmed by the electrograms. Gating is typically performed while the mapping catheter is in the coronary sinus.

**Figure 1:**
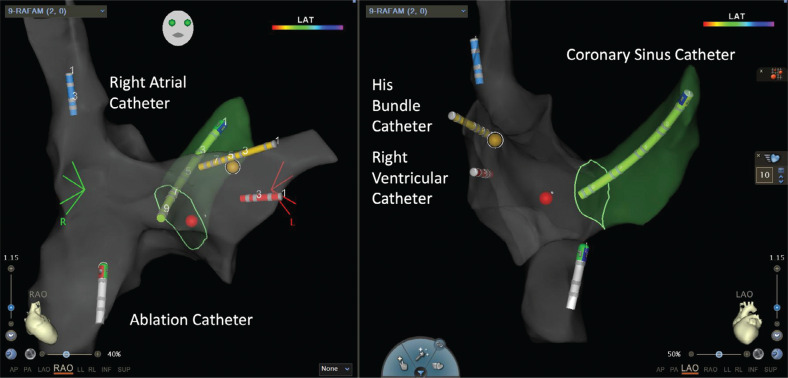
Electroanatomic map of the right atrium, superior vena cava, inferior vena cava, His bundle, and coronary sinus in a patient undergoing ablation of the slow pathway.

In supraventricular tachycardia ablation cases, the DECANAV catheter is kept in the coronary sinus and additional catheters are advanced into the right atrium, right ventricle, and His-bundle region. Should a right-sided arrhythmia circuit be identified, ablation can then be performed using the existing map **(Figure 1)**.

**Figure 2:**
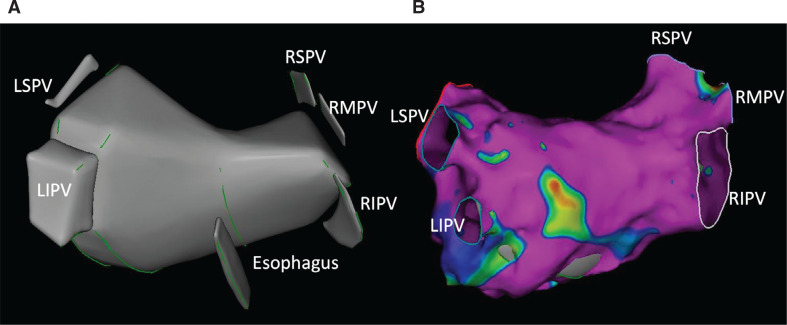
**(A)** An intracardiac echocardiography shell of the left atrium and its structures, which was used as a reference to construct **(B)** an electroanatomical map using the multipolar catheter after the transseptal puncture. *Abbreviations:* LIPV, left inferior pulmonary vein; LSPV, left superior pulmonary vein; RIPV, right inferior pulmonary vein; RMPV, right middle pulmonary vein; RSPV, right superior pulmonary vein.

In atrial fibrillation cases, the PentaRay catheter is withdrawn and a decapolar catheter is advanced into the coronary sinus, as guided by the previously created map. An ICE catheter is introduced into the right atrium while monitoring its tip on the electroanatomic map and ensuring intraluminal positioning in the IVC, which might require a slight clockwise rotation or anterior deflection. An ultrasound shell of the left atrial structures and esophagus is then made, starting in the home view followed by progressive clockwise rotation **(Figure 2A)**. To check for a baseline pericardial effusion, the ICE catheter is advanced into the right ventricle by going back to the home view, where the tricuspid valve can be seen, and performing an anterior tilt. The catheter is then advanced while maintaining septal positioning, and the left ventricle can be visualized by performing a clockwise rotation. We also typically use the ICE catheter for other left-sided and atrial flutter ablation cases and to ensure the absence of a post-procedural pericardial effusion.

## Esophageal probe insertion

**Figure 3:**
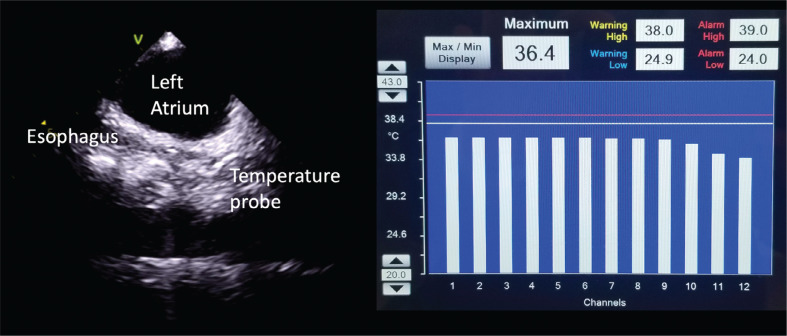
Fluoroless visualization of a multipoint, sinusoidal esophageal temperature probe (CIRCA S-CATH) using intracardiac echocardiography. The temperature spread can confirm the proper insertion depth; the esophageal temperature is lower on the proximal electrodes (10–12) closer to the trachea above the left atrium.

The esophagus can be visualized with ICE and the course depicted on the electroanatomic map. We typically use a sinusoidal multipoint temperature probe (CIRCA S-CATH; CIRCA Scientific, Englewood, CO, USA). A stylet is inserted to straighten the catheter, which should then be easily advanced. However, should resistance be encountered, the probe should be withdrawn and then re-advanced in a slightly different orientation. The probe can then be seen as it enters the esophagus **(Video 1)**, and the temperature spread is continuously monitored **(Figure 3)**. Advancement should be continued until the proximal electrodes (10–12) display a slightly lower temperature, which indicates that they are at the level of the trachea, and this should be a satisfactory position as it would span the length of the left atrium. The stylet is then withdrawn, and the probe is secured in place.

## Transseptal access

**Figure 4:**
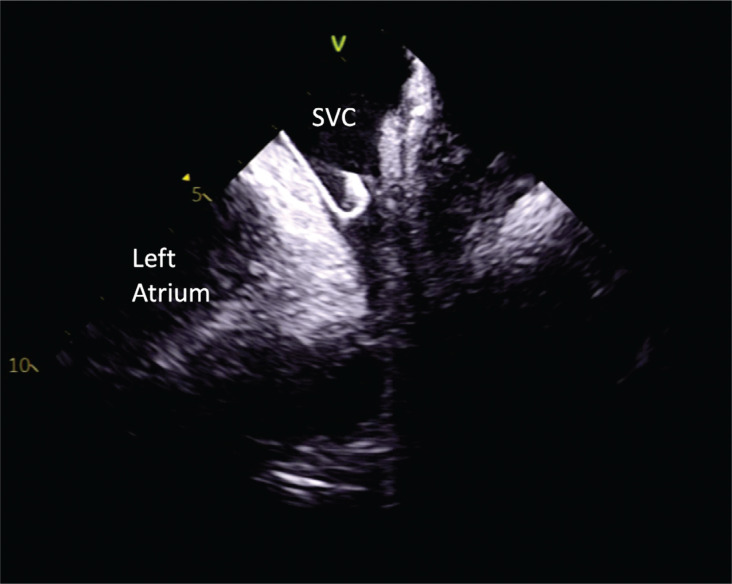
Intracardiac echocardiography image showing the J-tip wire in the superior vena cava. *Abbreviation:* SVC, superior vena cava.

**Figure 5:**
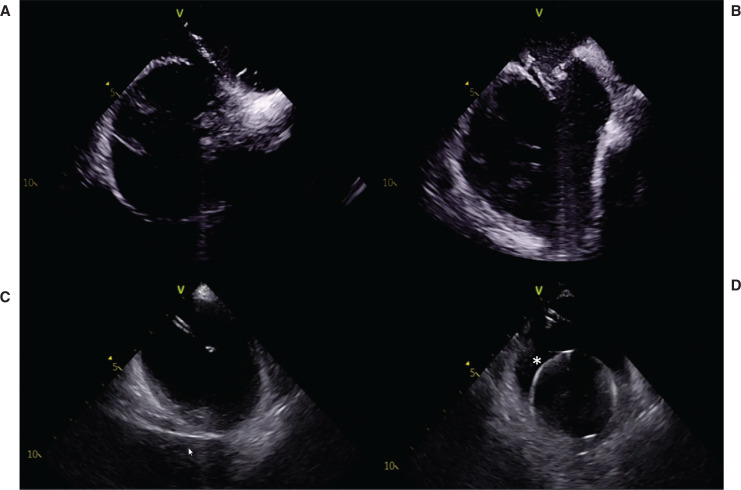
Intracardiac echocardiography showing the steps for a successful transseptal puncture. The transseptal system (sheath, dilator, and needle) is gradually withdrawn while maintaining the needle in the 4 o’clock position. A drop is typically felt after **(A)** the superior limbus of the interatrial septum has been reached, and another one is noted at the level of the fossa ovalis. **B:** The needle is then advanced to ensure “tenting” as it is exposed. Once the transseptal puncture has been performed, left atrial pressure is obtained to ensure safe placement. The needle is then held in place while **(C)** the dilator is advanced over it before **(D)** being exchanged for a ProTrack pigtail wire, as indicated by the asterisk. The arrow in **(C)** points to the aorta.

This is the most challenging step upon transitioning to a completely fluoroless workflow. We start by placing the ICE catheter in the SVC. The previously created electroanatomic map is used as a guide and, sometimes, a slight posterior tilt is needed. Once in the SVC, a posterior and right tilt is applied, and a good view showing the right atrial appendage as well as the main body of the SVC should be obtained. A J-tip wire is then advanced into the SVC and can be visualized as it enters **(Figure 4, Video 2)**. The wire should be moving freely, and attention is paid to avoid advancement into the right atrial appendage. The dilator and the transseptal sheath are then advanced over the wire and can also be visualized on ICE. The dilator is held in place while the sheath is advanced about 2 finger-widths over it. Saline can be injected to confirm correct positioning. The transseptal needle is then advanced until about 2 finger-widths from the dilator. The sheath is then brought back into the dilator and locked in position. The ICE catheter is subsequently brought back to the neutral position, and the interatrial septum should be visualized. The transseptal system (sheath, dilator, and needle) is gradually withdrawn while maintaining the needle in the 4 o’clock position. A drop is typically felt after the superior limbus of the interatrial septum has been reached, and another one is noted at the level of the fossa ovalis **(Figure 5A, Video 3)**. The ICE catheter can then be gradually withdrawn with gentle clockwise and counterclockwise rotations applied to confirm safe positioning of the dilator and sheath. The needle is then advanced with continued ICE to ensure “tenting” as it is exposed **(Figure 5B, Video 4)**. An NRG Transseptal Needle (Baylis Medical, Montreal, Canada) is typically used. Once the transseptal puncture has been performed, left atrial pressure is obtained to ensure safe placement. The needle is then held in place while the dilator is advanced over it **(Figure 5C)** before being exchanged for a ProTrack pigtail wire (Baylis Medical), which is easily visualized on ICE. The sheath is then advanced into the left atrium, and the wire and the dilator are removed **(Figure 5D, Video 5)**.

At times, anatomy can be challenging, and the J-tip wire is not clearly visualized as it is advanced into the SVC. In such cases, an alternative technique is used. The J-tip wire and the dilator are removed and exchanged for the PentaRay catheter, which is advanced into the SVC. We keep advancing the sheath over the catheter until the proximal poles are blacked out on the electroanatomic map **(Video 6)**, which should give us an indication about the exact level of the sheath as shown on the map. The sheath is then left in the SVC, and the PentaRay catheter is exchanged for the dilator and the needle. Additionally, there are several sheaths that can be visualized on the mapping system and would enable easier advancement into the SVC. The transseptal puncture can then be performed in the same fashion.

## Left atrial ablation

### Radiofrequency ablation

Two transseptal punctures are performed as described earlier. Alternatively, a single transseptal approach can be utilized. Once the initial transseptal access is made, a ProTrack pigtail wire is inserted into the left atrium. The transseptal sheath and its dilator are withdrawn into the right atrium. The transseptal access site is visualized on ICE and marked on the electroanatomic map. The ablation catheter can then be used to cross the septum, under ICE and electroanatomic guidance, before being introduced into one of the left-sided pulmonary veins and used as a rail to advance the long sheath into the left atrium. Thereafter, the first transseptal sheath can be re-advanced into the left atrium over the ProTrack pigtail wire and under ICE guidance. The PentaRay catheter is used to construct a left atrial electroanatomic map, and the ultrasound shell can be used as a guide to ensure all left atrial structures have been covered **(Figure 2B)**. Using a steerable sheath that can be visualized on the mapping system helps to stabilize the ablation catheter without exerting continuous manual pressure on the controls. The ICE catheter can also be used to assess the relationship between the left atrial wall and mapping and ablation catheters. The proximity of the phrenic nerve can be checked by using high-output pacing from the ablation catheter in the anterior antrum of the right-sided pulmonary veins.

### Cryoablation

**Figure 6:**
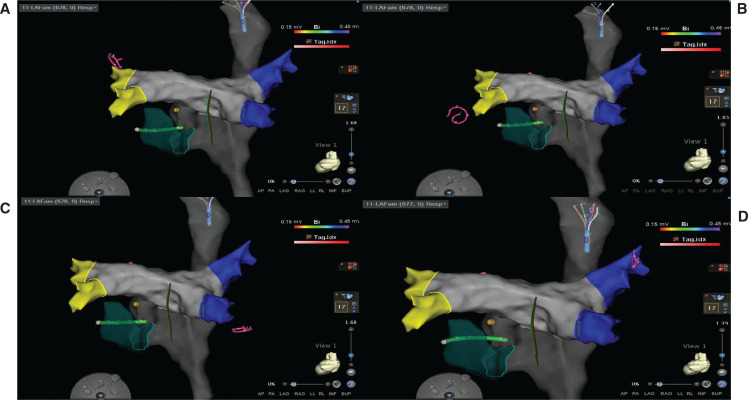
Proper Achieve catheter positioning using the electroanatomic map in the **(A)** left superior, **(B)** left inferior, **(C)** right inferior, and **(D)** right superior pulmonary veins.

**Figure 7:**
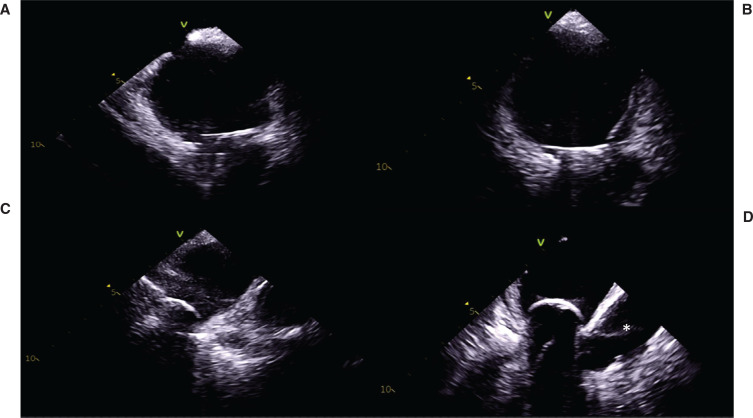
Proper cryoballoon positioning using intracardiac echocardiography in the **(A)** left superior, **(B)** left inferior, **(C)** right inferior, and **(D)** right superior pulmonary veins. Asterisk indicates the right pulmonary artery, which typically comes into view while visualizing the right superior pulmonary vein.

**Figure 8:**
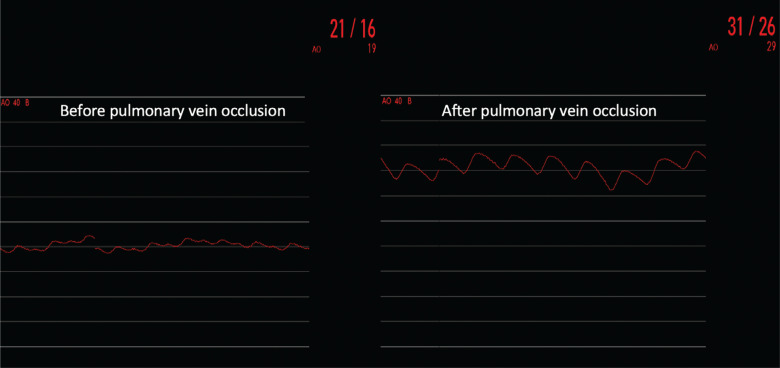
Pressure tracing during cryoablation: once the pulmonary vein is occluded, the pressure curve changes from a left atrial pressure curve to a pulmonary artery pressure curve.

Once the transseptal sheath has been advanced into the left atrium, as described earlier, the ProTrack pigtail wire is removed and exchanged for the multipolar catheter. An electroanatomic map of the left atrium and pulmonary veins is then generated, and the PentaRay catheter is removed and exchanged for the ProTrack pigtail wire. While maintaining constant ICE visualization of this wire in the left atrium, the transseptal sheath is exchanged for a FlexCath Advance system (Medtronic, Minneapolis, MN, USA) over the wire after progressive dilatation. The dilator and the sheath are advanced slowly until “tenting” can be visualized on ICE. Advancement is continued until the dilator is present in the left atrium. It is then held in place while gentle pressure is applied on the sheath until it is successfully advanced into the left atrium. The wire and the dilator are then removed and exchanged for the Arctic Front Advance Pro cryoballoon and Achieve catheter (Medtronic). The cryoballoon has markers on the shaft correlating with the front and back ends of the balloon. Once the initial marker is inside the sheath, the Achieve catheter is advanced and should be visualized on ICE as well as the electroanatomic map. Care should be taken while maneuvering the sheath in the left atrium, ensuring that the Achieve catheter is outside. To obtain coaxial alignment, the Achieve catheter is typically advanced into the upper branches of the superior pulmonary veins and lower branches of the inferior pulmonary veins **(Figure 6)**. The ideal antral position of the equatorial surface of the balloon is confirmed on ICE by the presence of the balloon’s proximal end within the left atrial cavity **(Figure 7)**. The sheath is advanced behind the back end of the balloon to obtain satisfactory closure. We start by isolating the left-sided pulmonary veins. Antral occlusion is confirmed by pressure monitoring **(Figure 8)** and Doppler echocardiography. Following the delivery of a circumferential lesion set around the left superior pulmonary vein antrum, the balloon is deflated and withdrawn while keeping the Achieve catheter in view. Under ICE and electroanatomic visualization, the sheath is deflected until the Achieve catheter is pointing directly toward the ostium of the left inferior pulmonary vein. It is then advanced deeply into the lower branch and used as a rail to advance the balloon, followed by the sheath. Typically, in this context, deflection of the balloon handle is also required for satisfactory closure.

To cannulate the right inferior pulmonary vein, we utilize a “hockey-stick” maneuver. First, the balloon is deflated and withdrawn until the Achieve catheter is exposed just beyond the tip of the sheath, which is then maximally deflected. The assembly is then gently withdrawn while applying clockwise rotation toward the right inferior pulmonary vein, as guided by ICE and the position of the Achieve catheter on the electroanatomic map, which has to be in view to ensure it is beyond the tip of the sheath and to avoid damage to the posterior wall. The Achieve catheter and the sheath should then typically fall into the right inferior pulmonary vein. We then advance the Achieve catheter into the lower branch and use it as a rail to advance the balloon. Before ablation is performed, the PentaRay catheter (or any other pacing catheter) is advanced into the SVC above the level of the pulmonary veins as guided by the electroanatomic map created earlier. Ablation is performed during continuous phrenic nerve capture with a manual assessment of diaphragmatic movement.

For cannulation of the right superior pulmonary vein, the ICE catheter is advanced superiorly with a slight posterior deflection until the vein is visualized. Typically, the right pulmonary artery will be in the same plane. After withdrawing the balloon from the right inferior pulmonary vein, deflection on the sheath is released, and it is then slowly turned clockwise. The Achieve catheter is then advanced into the upper branch of the right superior pulmonary vein and used as a rail to advance the balloon. The sheath is subsequently advanced until the back end of the balloon has been reached. Further clockwise rotation of the assembly is typically needed to obtain satisfactory closure. Antral ablation is then performed while monitoring for phrenic nerve injury, as described earlier.

## Right ventricular ablation

**Figure 9:**
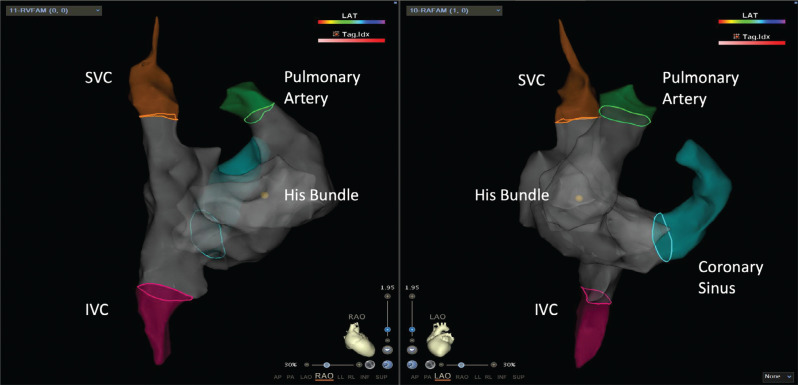
Electroanatomic map in a patient undergoing right ventricular outflow tract tachycardia ablation. *Abbreviations:* IVC, inferior vena cava; SVC, superior vena cava.

If outflow tract tachycardia or premature ventricular complexes (PVCs) are suspected, we start with a multipolar catheter to construct an electroanatomic map of the right atrium, right ventricle, and pulmonary artery **(Figure 9)**. In rotated or vertical hearts, the double angulation needed to reach and precisely map the superior outflow tract may be difficult with a single-curve catheter. In such cases, a steerable sheath would be helpful.

For focal arrhythmias, the PentaRay catheter can quickly identify the area of earliest activation. In this approach, compared to mapping with the ablation catheter, the electrode density is higher, and less force is needed to keep the electrodes on the tissue surface, decreasing the chance of catheter-induced PVCs. Intracardiac echocardiography can also be utilized to facilitate mapping and ablation.

## Left ventricular ablation

**Figure 10:**
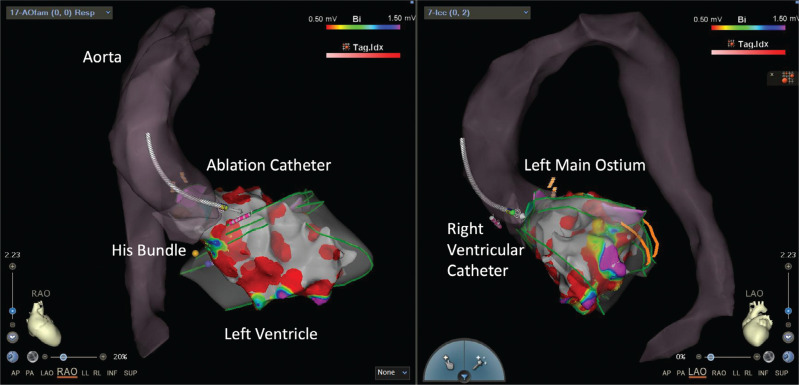
Electroanatomic map in a patient undergoing left ventricular tachycardia ablation. The map was guided by an ultrasound shell created earlier using intracardiac echocardiography.

Retrograde fluoroless access to the left ventricle can be obtained via the aorta. An ultrasound shell of the aortic root, aortic valve, and left ventricle is constructed by placing the ICE catheter in the right ventricle. Arterial access is obtained via a micropuncture needle set and under ultrasound guidance to ensure the common femoral artery is punctured just above its bifurcation. A long sheath is typically used to overcome any arterial tortuosity. Once the ablation catheter is advanced into the descending aorta via the sheath, force sensing is initialized. Crossing the aortic valve is most feasible with a tight curve on the ablation catheter; turning it around is typically performed in the upper portion of the descending aorta or aortic arch. Force should be monitored continuously during this maneuver to avoid engaging a side branch and exerting excessive pressure. Once the tip of the catheter is turned around, advancing it forward should be easy to achieve; however, when pulling back, care must be taken to monitor the force and change the curve as it may engage a side branch or a coronary artery. Should that be the case, advancing the catheter slightly forward and rotating it frees the tip **(Figure 10)**. For areas where the catheter cannot be advanced easily, ICE guidance to visualize the catheter tip and surrounding cardiac structures helps determine whether this area is occupied by an intraluminal structure or whether a different catheter orientation may be needed. A multipolar catheter can also be advanced in the same fashion for initial activation and voltage mapping before proceeding with ablation.

Arterial hemostasis is typically achieved by manual compression after reversal of anticoagulation. However, if an arterial closure device needs to be used, a peripheral angiogram should be obtained to ensure satisfactory anatomy, and this is a case where brief fluoroscopy after completion of ablation should be used for safety.

## Ablation in patients with implantable cardiac devices

**Figure 11:**
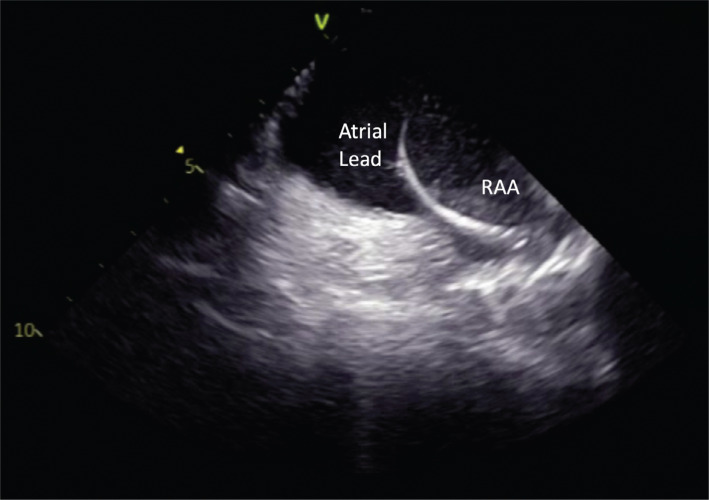
Intracardiac echocardiography image showing an atrial lead in the right atrial appendage. *Abbreviation:* RAA, right atrial appendage.

**Figure 12:**
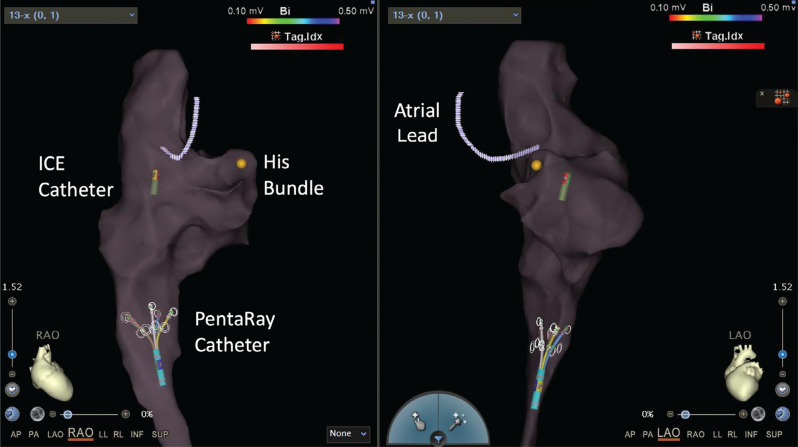
Incorporation of the course of the right atrial lead as shown on intracardiac echocardiography **(Figure 11)** into the electroanatomic map. *Abbreviation:* ICE, intracardiac echocardiography.

Patients with implantable cardiac devices pose a particular challenge to fluoroless ablation, especially if an atrial lead is present. We have been able to overcome this by using ICE to visualize the course of the leads **(Figure 11)** and incorporating it into the electroanatomic map of the respective chamber **(Figure 12)**.

## Conclusion

Fluoroless ablation of cardiac arrhythmias is safe and effective using the currently available technology. However, a proper understanding of cardiac anatomy, electroanatomic mapping techniques, and ICE is crucial before adopting such an approach.

## Figures and Tables

**Video 1:** Esophageal probe insertion.

**Video 2:** J-tip wire in the superior vena cava.

**Video 3:** Tenting on fossa.

**Video 4:** Transseptal puncture.

**Video 5:** Transseptal sheath advancement.

**Video 6:** Transseptal puncture using PentaRay catheter.
